# The Duties of Physicians in Regard to Insanity

**Published:** 1912-06

**Authors:** Francis E. Fronczak

**Affiliations:** Health Commissioner, Buffalo, N. Y.


### BUFFALO MEDICAL JOURNAL

Vol. Lxvii. JUNE, 1912. No. 11

### ORIGINAL COMMUNICATIONS

### The Duties of Physicians in Regard to Insanity.

By FRANCIS E. FRONCZAK. M. D., Health Commissioner, Buffalo, N. Y.

OUT of 200 or more sections with innumerable amendments, relative to the law on insanity, the following are the portions of the law which will be of general interest to physicians, in relation to the Health Department:

Any reputable physician, a graduate of an incorporated Medical College who has been in the active practice of his profession at least three years may become an examiner by filing with the State Commission at Albany a certified copy of the certificate of a judge of a court of record showing such qualifications in accordance with forms prescribed by the State Commission in Lunacy.

Duties of local officers in regard to their insane. All county superintendents of the poor, overseers of the poor and other city, town or county authorities, having duties to perform relating to the *insane poor* are charged with the duty of seeing that all *poor* and *indigent* insane persons within their respective municipalities are timely granted the necessary relief conferred by this chapter. The poor officers or authorities above specified shall notify the Health Officer of the city of any *poor* or *indigent* insane or apparently insane person within such municipality whom they know to be in need of relief conferred by this chapter. When so notified or when otherwise informed of such fact, the Health Officer shall see that proceedings are taken for the determination of his mental condition, and for his commitment to a state hospital. When notified or informed of any *poor* or *indigent* insane, or apparently insane person in need of relief conferred by this chapter, the Health Officer shall provide for the proper *care, treatment* and *nursing* of such person pending the determination of his mental condition and his commitment.

Sec. 80. Order for commitment of an insane person. A person alleged to be insane, and who is not in confinement on a criminal charge, may be committed to and confined in an institution for the custody and treatment of the insane, upon an order made by a judge of a court of record of the city or county, or a justice of the supreme court of the judicial district, in which the alleged insane person resides or may be, adjudging such person to be insane, upon a certificate of lunacy, made by two qualified medical examiners in lunacy, accompanied by a verified petition therefor, or upon such certificate and petition, and after a hearing to determine such question, as provided in this article. The commission shall prescribe and furnish blanks for such certificates and petitions, which shall be made only upon such blanks. An insane person shall be committed to a state hospital, a duly licensed institution for the insane, or the Matteawan State hospital, or to the care and custody of a relative or committee, as hereinafter provided. No idiot shall be committed to or confined in a state hospital. But any epileptic or feeble-minded person becoming insane may be committed as an insane person to a state hospital for custody and treatment therein.

Sec. 81. Physicians shall jointly make a final examination of the person alleged to be insane within ten days next before the granting of the order. The date of the certificate of lunacy shall be the date of such joint examination. Such certificate of lunacy shall be in the form prescribed by the commission, and shall contain the facts and circumstances upon which the judgment of the physician is based and show that the condition of the person examined is such as to require care and treatment in an institution for the care, custody and treatment of the insane.

Neither of such physicians shall be a relative of the person applying for the order or of the person alleged to be insane, or a manager, superintendent, proprietor, officer, stockholder, or have any pecuniary interest, directly or indirectly, or be an attending physician in the institution to which it is proposed to commit such person.

Sec. 82. Proceedings to determine the question of insanity. Any person with whom an alleged insane person may reside or at whose house he may be, or the father or mother, husband or wife, brother or sister, or the child of any such person, and any overseer of the poor of the town, and superintendent of the poor of the county in which any such person may be, may apply for such order, by presenting a verified petition containing a statement of the facts, upon which the allegation of insanity is based, and because of which the application for the order is made. Such petition shall be accompanied by the certificates of lunacy of the medical examiners, as prescribed in the preceding section. Notice of such application shall be served personally, at least one day before making such application, upon the person alleged to be insane, and if made by an overseer or superintendent of the poor, also upon the husband or wife, father or mother or next of kin of such alleged insane person, if there be any such known to be residing within the county, and if not, upon the person with whom such alleged insane person may reside, or at whose house he may be. The judge to whom the application is to be made may dispense with such personal service, or may direct substituted service to be made upon some person to be designated by him. He shall state in a certificate to be attached to the petition his reason for dispensing with personal service of such notice and if substituted service is directed, the name of the person to be served therewith.

The judge to whom such application is made may, if no demand is made for a hearing in behalf of the alleged insane person, proceed forthwith to determine the question of insanity, and if satisfied that the alleged insane person is insane, may immediately issue an order for the commitment of such person to an institution for the custody and treatment of the insane. If, however, it appears that such insane person is harmless and his relatives or a committee of his person are willing and able to properly care for him, at some place other than such institution, upon their written consent, the judge may order that he be placed in the care and custody of such relatives or such committee. Such judge may, in his discretion, require other proofs in addition to the petition and certificate of the medical examiners.

Sec. 84. Costs of commitment. The costs necessarily incurred in determining the question of the insanity of a poor or indigent person and in securing his admission into a state hospital, and the expense of providing proper clothing for such person, in accordance with the rules and regulations adopted by the commission, shall be a charge upon the town, city or county securing the commitment. Such costs shall include the fees allowed by the judge or justice ordering the commitment to the medical examiners. If the person sought to be committed is not a poor or indigent person, the costs of the proceedings to determine his insanity and to secure his commitment, as provided in this article, shall be a charge upon his estate, or shall be paid by the persons legally liable for his maintenance. If in such proceedings, the alleged insane person is determined not to be insane, the judge or justice may, in his discretion, charge the costs of the proceedings to the person making the application for an order of commitment, and judgment may be entered for the amount thereof and enforced by execution against such person.

LAW RELATIVE TO EMERGENCY CASES.

Notwithstanding the requirements of the law that an alleged insane person be duly committed by an order of the court, in a case where the condition of such person is such that it would be for his benefit to receive immediate care and treatment, or if he is dangerously insane so as to render it necessary for public safety that he be immediately confined, he shall be forthwith received by a state institution authorized by law to care for the insane. In such case such insane person shall be so received by such institution, and upon a petition made by the person authorized by this section to apply to a court for an order of commitment. By virtue of such certificate of lunacy and such petition such insane person may be retained in such institution for a period not to exceed five days. Prior to the expiration of such time an order for his commitment must be obtained in the manner provided by this section. The certificate of lunacy executed by such physicians must contain adequate reasons why the insane person should be immediately received in an institution for the insane for treatment. The superintendent or person in charge of any such institution may refuse to receive such insane person upon such certificate and petition, if in his judgment the reasons stated in the certificate are not sufficient, or the condition of the patient is not of such character, as to make it necessary that the patient should receive immediate treatment.

LAW RELATIVE TO VOLUNTARY PATIENTS.

The superintendent in charge of any state hospital for the insane (except Matteawan or Dannemora) may receive and retain therein any person suitable for care and treatment and who voluntarily makes written application therefor and whose mental condition is such as to render him competent to make such application. Such patient must give five (5) days notice in writing to the Superintendent before leaving the hospital.

